# Supplementary material for: Modelling Skylarks (Alauda arvensis) to Predict Impacts of Changes in Land Management and Policy: Development and Testing of an Agent-Based Model
Source: PLoS One. 2013 Jun 6;8(6):e65803. doi: 10.1371/journal.pone.0065803 (PMC3675089; doi:10.1371/journal.pone.0065803)
Supplement: Supporting Information S4 — The skylark ODdox as a zipped archive. (ZIP) [file pone.0065803.s004.zip › Skylark_ODdox/class_population___manager.html]

ALMaSS Skylark ODdox: Population\_Manager Class Reference


ALMaSS Skylark ODdox
 2.0


- Main Page
- Related Pages
- Classes
- Files

- Class List
- Class Index
- Class Hierarchy
- Class Members

Public Member Functions |
Public Attributes |
Protected Member Functions |
Protected Attributes

Population\_Manager Class Reference

Base class for all population managers.
More...

`#include <populationmanager.h>`

List of all members.

|  |  |
| --- | --- |
| Public Member Functions | |
| bool | BeginningOfMonth () |
| virtual void | BreedingPairsOutput (int) |
| virtual void | BreedingSuccessProbeOutput (double, int, int, int, int, int, int, int) |
| virtual void | Catastrophe (int) |
| bool | CheckXY (int l, int i) |
|  | Debug method to test for out of bounds coordinates. |
| void | CloseTheCIPEGridOutputProbe () |
| void | CloseTheMonthlyRipleysOutputProbe () |
| void | CloseTheReallyBigOutputProbe () |
| void | CloseTheRipleysOutputProbe () |
| virtual void | DisplayLocations () |
| TAnimal \* | FindClosest (int x, int y, unsigned Type) |
| virtual void | FledgelingProbeOutput (int, int) |
| virtual void | GeneticsResultsOutput (FILE \*, unsigned) |
| virtual void | ImpactedProbe () |
| void | ImpactProbeReport (int a\_Time) |
| bool | IsLast (unsigned listindex) |
| void | LamdaBirth (int x, int y) |
| void | LamdaBirth (int x, int y, int z) |
| void | LamdaClear () |
| void | LamdaDeath (int x, int y) |
| void | LamdaDumpOutput () |
| void | LOG (const char \*fname) |
| virtual bool | OpenTheBreedingPairsProbe () |
| virtual bool | OpenTheBreedingSuccessProbe () |
| bool | OpenTheCIPEGridOutputProbe () |
| virtual bool | OpenTheFledgelingProbe () |
| bool | OpenTheMonthlyRipleysOutputProbe () |
| bool | OpenTheReallyBigProbe () |
| bool | OpenTheRipleysOutputProbe () |
|  | Population\_Manager (Landscape \*L) |
| virtual float | Probe (int ListIndex, probe\_data \*p\_TheProbe) |
| int | ProbeFileInput (char \*p\_Filename, int p\_ProbeNo) |
| char \* | ProbeReport (int a\_time) |
| char \* | ProbeReportTimed (int a\_time) |
| virtual void | Run (int NoTSteps) |
| void | SetNoProbes (int a\_pn) |
| char \* | SpeciesSpecificReporting (int a\_species, int a\_time) |
| virtual int | SupplyCovPosx (int) |
| virtual int | SupplyCovPosy (int) |
| unsigned | SupplyListIndexSize () |
| const char \* | SupplyListName (int i) |
| int | SupplyListNameLength () |
| unsigned | SupplyListSize (unsigned listindex) |
| virtual void | SupplyLocXY (unsigned listindex, unsigned j, int &x, int &y) |
| virtual int | SupplyPegPosx (int) |
| virtual int | SupplyPegPosy (int) |
| int | SupplySimH () |
| int | SupplySimW () |
| int | SupplyState (unsigned listindex, unsigned j) |
| IntArray100 \* | SupplyStateList () |
| const char \* | SupplyStateNames (int i) |
| unsigned | SupplyStateNamesLength () |
| int | SupplyStepSize () |
| virtual int | TheBreedingFemalesProbe (int) |
| virtual int | TheBreedingSuccessProbe (int &, int &, int &, int &, int &, int &) |
| virtual void | TheCIPEGridOutputProbe () |
| virtual int | TheFledgelingProbe () |
| virtual void | TheGeneticProbe (unsigned, int, unsigned &) |
| virtual void | TheNWordOutputProbe () |
| virtual void | TheReallyBigOutputProbe () |
| virtual void | TheRipleysOutputProbe (FILE \*a\_prb) |
| virtual | ~Population\_Manager (void) |

|  |  |
| --- | --- |
| Public Attributes | |
| AlleleFreq \* | AlFreq |
| unsigned | Counts [10][100] |
| int | IndexArrayX [5][10000] |
| MyDialog \* | m\_MainForm |
| char | m\_SimulationName [255] |
| Landscape \* | m\_TheLandscape |
| bool | ProbesSet |
| int | SimH |
| unsigned | SimHH |
| int | SimW |
| unsigned | SimWH |
| probe\_data \* | TheProbe [100] |

|  |  |
| --- | --- |
| Protected Member Functions | |
| virtual void | Catastrophe () |
| virtual void | DoAfter () |
| virtual void | DoAlmostLast () |
| virtual void | DoBefore () |
| virtual void | DoFirst () |
| virtual void | DoLast () |
| void | NWordOutput () |
|  | The output section for the NWord probe.  . |
| void | Shuffle (unsigned Type) |
| void | Shuffle\_or\_Sort (unsigned Type) |
| void | SortState (unsigned Type) |
| void | SortStateR (unsigned Type) |
| void | SortX (unsigned Type) |
| void | SortXIndex (unsigned Type) |
| void | SortY (unsigned Type) |
| virtual bool | StepFinished () |

|  |  |
| --- | --- |
| Protected Attributes | |
| unsigned | BeforeStepActions [10] |
| FILE \* | CIPEGridOutputPrb |
| FILE \* | CIPEGridOutputPrbB |
| long int | lamdagrid [2][257][257] |
| FILE \* | m\_AlleleFreqsFile |
| int | m\_catastrophestartyear |
| int \* | m\_cgridcount |
| int | m\_cgridcountwidth |
| int | m\_cipegridsize |
| FILE \* | m\_EasyPopRes |
| FILE \* | m\_GeneticsFile |
| int \* | m\_gridcount [4] |
| int | m\_gridcountsize [4] |
| int | m\_gridcountwidth [4] |
| unsigned | m\_ListNameLength |
| const char \* | m\_ListNames [10] |
| int | m\_NoProbes |
| int | m\_StepSize |
| FILE \* | NWordOutputPrb |
| FILE \* | ReallyBigOutputPrb |
| FILE \* | RipleysOutputPrb |
| FILE \* | RipleysOutputPrb1 |
| FILE \* | RipleysOutputPrb10 |
| FILE \* | RipleysOutputPrb11 |
| FILE \* | RipleysOutputPrb12 |
| FILE \* | RipleysOutputPrb2 |
| FILE \* | RipleysOutputPrb3 |
| FILE \* | RipleysOutputPrb4 |
| FILE \* | RipleysOutputPrb5 |
| FILE \* | RipleysOutputPrb6 |
| FILE \* | RipleysOutputPrb7 |
| FILE \* | RipleysOutputPrb8 |
| FILE \* | RipleysOutputPrb9 |
| IntArray100 | StateList |
| const char \* | StateNames [100] |
| unsigned | StateNamesLength |
| FILE \* | TestFile |
| FILE \* | TestFile2 |
| vector< TListOfAnimals > | TheArray |

---

## Detailed Description

Base class for all population managers.

The core of the handling of animal populations. All time-step code and most input/output is handled by this class and its descendents. This class effectively implements a state machine to facilitate simulation of animal behaviours and handle potential issues with concurrency. The PopulationManager class is never instantiated but must be used by deriving a descendent class.

---

## Constructor & Destructor Documentation

|  |  |  |  |  |  |
| --- | --- | --- | --- | --- | --- |
| Population\_Manager::Population\_Manager | ( | Landscape \* | *L* | ) |  |

Constructor for the Population\_Manager class

References cfg\_CipeGridSize, cfg\_RipleysOutputMonthly\_used, CfgInt::value(), and CfgBool::value().

{

// Keep a pointer to the landscape this population is in

m\_TheLandscape = L;

// create 10 empty arrays as default, excess can be removed in descendent classes

TListOfAnimals alist;

TheArray.insert( TheArray.end(), 10, alist );

// Set the simulation bounds

SimH = m\_TheLandscape->SupplySimAreaHeight();

SimW = m\_TheLandscape->SupplySimAreaWidth();

SimHH = m\_TheLandscape->SupplySimAreaHeight()/2;

SimWH = m\_TheLandscape->SupplySimAreaWidth()/2;

// Set default BeforeStepActions

for ( int i = 0; i < 10; i++ ) m\_ListNames[i] = "Unknown";

// Set default BeforeStepActions

for ( int i = 0; i < 10; i++ ) BeforeStepActions[ i ] = 0;

// modify this if they need to in descendent classes

StateNamesLength = 0; // initialise this variable.

m\_catastrophestartyear=-1; // Default is don't do this

#ifdef \_\_LAMBDA\_RECORD

ofstream fout("LambdaGridOuput.txt", ios\_base::out); // open for writing

fout.close();

LamdaClear();

#endif

if ( cfg\_RipleysOutputMonthly\_used.value() ) {

OpenTheMonthlyRipleysOutputProbe();

}

m\_cipegridsize = cfg\_CipeGridSize.value();

m\_cgridcountwidth = SimW/m\_cipegridsize;

m\_cgridcount = new int[ m\_cgridcountwidth \* m\_cgridcountwidth ];

m\_gridcountsize[0] = 50;

m\_gridcountsize[1] = 100;

m\_gridcountsize[2] = 200;

m\_gridcountsize[3] = 500;

m\_gridcountwidth[0] = SimW/50;

m\_gridcountwidth[1] = SimW/100;

m\_gridcountwidth[2] = SimW/200;

m\_gridcountwidth[3] = SimW/500;

m\_gridcount[0] = new int[ m\_gridcountwidth[0] \* m\_gridcountwidth[0] ];

m\_gridcount[1] = new int[ m\_gridcountwidth[1] \* m\_gridcountwidth[1] ];

m\_gridcount[2] = new int[ m\_gridcountwidth[2] \* m\_gridcountwidth[2] ];

m\_gridcount[3] = new int[ m\_gridcountwidth[3] \* m\_gridcountwidth[3] ];

}

|  |  |  |  |  |  |
| --- | --- | --- | --- | --- | --- |
| Population\_Manager::~Population\_Manager | ( | void |  | ) |  |

virtual

Destructor for the Population\_Manager class

References cfg\_ReallyBigOutput\_used, cfg\_RipleysOutput\_used, cfg\_RipleysOutputMonthly\_used, and CfgBool::value().

{

// clean-up

for ( unsigned i = 0; i < TheArray.size(); i++ ) {

// if objects in the list need to be deleted:

for ( unsigned j = 0; j < TheArray[ i ].size(); j++ ) {

delete TheArray[ i ] [ j ];

}

// empty the array

TheArray[ i ].clear();

// --

}

if ( cfg\_RipleysOutput\_used.value() ) {

CloseTheRipleysOutputProbe();

}

if ( cfg\_RipleysOutputMonthly\_used.value() ) {

CloseTheMonthlyRipleysOutputProbe();

}

if ( cfg\_ReallyBigOutput\_used.value() ) {

CloseTheReallyBigOutputProbe();

}

for (int d=0; d<4; d++) delete [] m\_gridcount[d];

delete [] m\_cgridcount;

}

---

## Member Function Documentation

|  |  |  |  |  |
| --- | --- | --- | --- | --- |
| bool Population\_Manager::BeginningOfMonth | ( |  | ) |  |

Is it the first day of the month?

References cfg\_DayInMonth, and CfgInt::value().

{

if ( m\_TheLandscape->SupplyDayInMonth() == cfg\_DayInMonth.value() ) return true;

return false;

}

|  |  |  |  |  |  |
| --- | --- | --- | --- | --- | --- |
| virtual void Population\_Manager::BreedingPairsOutput | ( | int |  | ) |  |

inlinevirtual

Reimplemented in Skylark\_Population\_Manager.

{

}

|  |  |  |  |
| --- | --- | --- | --- |
| virtual void Population\_Manager::BreedingSuccessProbeOutput | ( | double | , |
|  |  | int | , |
|  |  | int | , |
|  |  | int | , |
|  |  | int | , |
|  |  | int | , |
|  |  | int | , |
|  |  | int |  |
|  | ) |  |  |

inlinevirtual

Reimplemented in Skylark\_Population\_Manager.

{

}

|  |  |  |  |  |  |
| --- | --- | --- | --- | --- | --- |
| virtual void Population\_Manager::Catastrophe | ( | int |  | ) |  |

inlinevirtual

{

}

|  |  |  |  |  |  |
| --- | --- | --- | --- | --- | --- |
| void Population\_Manager::Catastrophe | ( | void |  | ) |  |

protectedvirtual

This method MUST be overidden in descendent classes if this functionality is does not match with the animals requirements

Reimplemented in Skylark\_Population\_Manager.

{

return;

}

|  |  |  |  |
| --- | --- | --- | --- |
| bool Population\_Manager::CheckXY | ( | int | *l*, |
|  |  | int | *i* |
|  | ) |  |  |

Debug method to test for out of bounds coordinates.

{

if (TheArray[l][i]->Supply\_m\_Location\_x() > 10000) return true;

if (TheArray[l][i]->Supply\_m\_Location\_y() > 10000) return true;

if (TheArray[l][i]->Supply\_m\_Location\_x() < 0) return true;

if (TheArray[l][i]->Supply\_m\_Location\_y() < 0) return true;

return false;

}

|  |  |  |  |  |
| --- | --- | --- | --- | --- |
| void Population\_Manager::CloseTheCIPEGridOutputProbe | ( |  | ) |  |

close the probe

{

if ( CIPEGridOutputPrb != NULL )

fclose( CIPEGridOutputPrb );

fclose( CIPEGridOutputPrbB );

fclose ( NWordOutputPrb );

}

|  |  |  |  |  |
| --- | --- | --- | --- | --- |
| void Population\_Manager::CloseTheMonthlyRipleysOutputProbe | ( |  | ) |  |

close the monthly probes

{

fclose( RipleysOutputPrb1 );

fclose( RipleysOutputPrb2 );

fclose( RipleysOutputPrb3 );

fclose( RipleysOutputPrb4 );

fclose( RipleysOutputPrb5 );

fclose( RipleysOutputPrb6 );

fclose( RipleysOutputPrb7 );

fclose( RipleysOutputPrb8 );

fclose( RipleysOutputPrb9 );

fclose( RipleysOutputPrb10 );

fclose( RipleysOutputPrb11 );

fclose( RipleysOutputPrb12 );

}

|  |  |  |  |  |
| --- | --- | --- | --- | --- |
| void Population\_Manager::CloseTheReallyBigOutputProbe | ( |  | ) |  |

close the probe

{

if ( ReallyBigOutputPrb != 0 )

fclose( ReallyBigOutputPrb );

ReallyBigOutputPrb=0;

}

|  |  |  |  |  |
| --- | --- | --- | --- | --- |
| void Population\_Manager::CloseTheRipleysOutputProbe | ( |  | ) |  |

close the probe

References cfg\_RipleysOutputMonthly\_used, and CfgBool::value().

{

if ( cfg\_RipleysOutputMonthly\_used.value() )

fclose( RipleysOutputPrb );

RipleysOutputPrb=NULL;

}

|  |  |  |  |  |
| --- | --- | --- | --- | --- |
| void Population\_Manager::DisplayLocations | ( |  | ) |  |

virtual

Not currently used

{

/\* for (unsigned j=0; j<TheArray[listindex].size(); j++) { int x=TheArray[listindex][j]->Supply\_m\_Location\_x();

int y=TheArray[listindex][j]->Supply\_m\_Location\_y(); m\_MainForm->Spot(listindex,x,y); } \*/

/\* // Skylark below if (m\_MainForm->RadioButton2->Checked) {

switch ((TTypesOfSkState)TheArray[listindex][j]->WhatState()) { case toss\_MFloating: case toss\_MFlocking:

case toss\_FFloating: case toss\_FFlocking: break; default: int x=TheArray[listindex][j]->Supply\_m\_Location\_x();

int y=TheArray[listindex][j]->Supply\_m\_Location\_y(); m\_MainForm->Spot(listindex,x,y); break; } }

\*/

/\* Partridge This code will only show coveys with chicks switch ((Partridge\_State)AManager->SupplyState(listindex,j)) {

case pars\_MCaringForYoung: case pars\_FCaringForYoung: AManager->SupplyLocXY(listindex,j,x,y); Form6->Spot(listindex,x,y);

break; default: break; } \*/

/\* This code shows all covey pegs & positions if (listindex==4) // Coveys { int x = SupplyPegPosx(j);

int y = SupplyPegPosy(j); m\_MainForm->Spot(1,x,y); x = SupplyCovPosx(j); y = SupplyCovPosy(j); m\_MainForm->Spot(2,x,y); }

#else int x=TheArray[listindex][j]->Supply\_m\_Location\_x(); int y=TheArray[listindex][j]->Supply\_m\_Location\_y();

m\_MainForm->Spot(listindex,x,y); \*/

}

|  |  |  |  |  |
| --- | --- | --- | --- | --- |
| void Population\_Manager::DoAfter | ( |  | ) |  |

protectedvirtual

Can be used in descendent classes

{

//TODO: Add your source code here

}

|  |  |  |  |  |
| --- | --- | --- | --- | --- |
| void Population\_Manager::DoAlmostLast | ( |  | ) |  |

protectedvirtual

Can be used in descendent classes

{

//TODO: Add your source code here

}

|  |  |  |  |  |
| --- | --- | --- | --- | --- |
| void Population\_Manager::DoBefore | ( |  | ) |  |

protectedvirtual

Can be used in descendent classes

{

}

|  |  |  |  |  |  |
| --- | --- | --- | --- | --- | --- |
| void Population\_Manager::DoFirst | ( | void |  | ) |  |

protectedvirtual

Can be used in descendent classes

Reimplemented in Skylark\_Population\_Manager.

{

}

|  |  |  |  |  |
| --- | --- | --- | --- | --- |
| void Population\_Manager::DoLast | ( |  | ) |  |

protectedvirtual

Collects some data to describe the number of animals in each state at the end of the day

{

#ifdef \_\_UNIX\_\_

for ( unsigned i = 0; i < 100; i++ ) {

StateList.n[ i ] = 0;

}

#else

// Zero results

for ( unsigned listindex = 0; listindex < m\_ListNameLength; listindex++ ) {

for ( unsigned i = 0; i < 100; i++ ) {

Counts[ listindex ] [ i ] = 0;

}

}

#endif

// How many animals in each state?

for ( unsigned listindex = 0; listindex < TheArray.size(); listindex++ ) {

unsigned size = (unsigned) TheArray[ listindex ].size();

for ( unsigned j = 0; j < size; j++ ) {

#ifdef \_\_UNIX\_\_

StateList.n[ TheArray[ listindex ] [ j ]->WhatState() ] ++;

#else

Counts[ listindex ] [ TheArray[ listindex ] [ j ]->WhatState() ] ++;

#endif

}

}

}

|  |  |  |  |
| --- | --- | --- | --- |
| TAnimal \* Population\_Manager::FindClosest | ( | int | *x*, |
|  |  | int | *y*, |
|  |  | unsigned | *Type* |
|  | ) |  |  |

Finds the closest individual to an x,y point

References TAnimal::Supply\_m\_Location\_x().

{

int distance = 100000000;

TAnimal \* TA = NULL;

int dx, dy, d;

for ( unsigned j = 0; j < TheArray[ Type ].size(); j++ ) {

dx = TheArray[ Type ] [ j ]->Supply\_m\_Location\_x();

dy = TheArray[ Type ] [ j ]->Supply\_m\_Location\_y();

dx = ( dx - x );

dx \*= dx;

dy = ( dy - y );

dy \*= dy;

d = dx + dy;

if ( d < distance ) {

distance = d;

TA = TheArray[ Type ] [ j ];

}

}

return TA;

}

|  |  |  |  |
| --- | --- | --- | --- |
| virtual void Population\_Manager::FledgelingProbeOutput | ( | int | , |
|  |  | int |  |
|  | ) |  |  |

inlinevirtual

Reimplemented in Skylark\_Population\_Manager.

{

}

|  |  |  |  |
| --- | --- | --- | --- |
| virtual void Population\_Manager::GeneticsResultsOutput | ( | FILE \* | , |
|  |  | unsigned |  |
|  | ) |  |  |

inlinevirtual

{

}

|  |  |  |  |  |
| --- | --- | --- | --- | --- |
| void Population\_Manager::ImpactedProbe | ( |  | ) |  |

virtual

Special pesticide related probe. Overidden in descendent classes

{

}

|  |  |  |  |  |  |
| --- | --- | --- | --- | --- | --- |
| void Population\_Manager::ImpactProbeReport | ( | int | *a\_Time* | ) |  |

Special probe

References BeginningOfMonth().

{

for ( int ProbeNo = 0; ProbeNo < 1; ProbeNo++ ) {

// See if we need to record/update this one

// if time/months/years ==0 or every time

if ( ( TheProbe[ ProbeNo ]->m\_ReportInterval == 3 )

|| ( ( TheProbe[ ProbeNo ]->m\_ReportInterval == 2 ) && ( BeginningOfMonth() ) )

|| ( ( TheProbe[ ProbeNo ]->m\_ReportInterval == 1 ) && ( a\_Time % 365 == 0 ) ) ) {

ImpactedProbe();

}

}

}

|  |  |  |  |  |  |
| --- | --- | --- | --- | --- | --- |
| bool Population\_Manager::IsLast | ( | unsigned | *listindex* | ) |  |

inline

References TheArray.

{

if ( TheArray[ listindex ].size() > 1 ) return false; else

return true;

}

|  |  |  |  |
| --- | --- | --- | --- |
| void Population\_Manager::LamdaBirth | ( | int | *x*, |
|  |  | int | *y* |
|  | ) |  |  |

inline

References lamdagrid.

{

lamdagrid[0][x / \_\_lgridsize][y / \_\_lgridsize ]++;

}

|  |  |  |  |
| --- | --- | --- | --- |
| void Population\_Manager::LamdaBirth | ( | int | *x*, |
|  |  | int | *y*, |
|  |  | int | *z* |
|  | ) |  |  |

inline

References lamdagrid.

{

lamdagrid[0][x / \_\_lgridsize][y / \_\_lgridsize ]+=z;

}

|  |  |  |  |  |
| --- | --- | --- | --- | --- |
| void Population\_Manager::LamdaClear | ( |  | ) |  |

inline

References lamdagrid.

{

for (int i=0; i<257; i++ ) {

for (int j=0; j<257; j++) {

lamdagrid[0][i][j]=0;

lamdagrid[1][i][j]=0;

}

}

}

|  |  |  |  |
| --- | --- | --- | --- |
| void Population\_Manager::LamdaDeath | ( | int | *x*, |
|  |  | int | *y* |
|  | ) |  |  |

inline

References lamdagrid.

{

// inlined for speed

lamdagrid[1][x / \_\_lgridsize][y / \_\_lgridsize ]++;

}

|  |  |  |  |  |
| --- | --- | --- | --- | --- |
| void Population\_Manager::LamdaDumpOutput | ( |  | ) |  |

Special probe

{

ofstream fout("LambdaGridOuput.txt", ios\_base::app); // open for writing

for (int i=0; i<257; i++ ) {

for (int j=0; j<257; j++) {

fout << lamdagrid[0][i][j] << "\t" << lamdagrid[1][i][j] << endl;

}

}

//fout << "NEXT" << endl;

fout.close();

}

|  |  |  |  |  |  |
| --- | --- | --- | --- | --- | --- |
| void Population\_Manager::LOG | ( | const char \* | *fname* | ) |  |

Debug function used to log whatever is needed - this is just a place to write whatever is needed at the time - so contents vary

References AnimalPosition::m\_x, and AnimalPosition::m\_y.

{

FILE \* PFile = fopen(fname, "w" );

if (PFile) {

m\_TheLandscape->Warn("PopulationManager::LOG - Could not open file ",fname);

exit(0);

}

AnimalPosition AP;

for ( unsigned listindex = 0; listindex < TheArray.size(); listindex++ ) {

#ifndef \_\_BCB\_\_

fprintf( PFile, "%s :\n", m\_ListNames[ listindex ] );

#else

fprintf( PFile, "%s :\n", m\_ListNames[ listindex ].c\_str() );

#endif

for ( unsigned j = 0; j < TheArray[ listindex ].size(); j++ ) {

AP = TheArray[ listindex ] [ j ]->SupplyPosition();

fprintf( PFile, "%i %i %i\n", j, AP.m\_x, AP.m\_y );

}

}

fclose( PFile );

}

|  |  |  |  |  |
| --- | --- | --- | --- | --- |
| void Population\_Manager::NWordOutput | ( |  | ) |  |

protected

The output section for the NWord probe.  
.

```
                         A common output stub for the NWord output probe. Specialist counting for each species
```

occurs as part of the CIPE grid probe, then this method deals with the final output.

Referenced by Skylark\_Population\_Manager::TheNWordOutputProbe().

{

int Counted[4];

int totalcells[4];

int OccupiedCells[4];

for (int cellwidth=0; cellwidth<4; cellwidth++) {

Counted[cellwidth] = 0;

totalcells[cellwidth] = m\_gridcountwidth[cellwidth] \* m\_gridcountwidth[cellwidth];

OccupiedCells[cellwidth] = 0;

for (int i=0; i<m\_gridcountwidth[cellwidth]; i++) {

for (int j=0; j<m\_gridcountwidth[cellwidth]; j++) {

int res=m\_gridcount[cellwidth][i+j\*m\_gridcountwidth[cellwidth]];

Counted[cellwidth] += res;

if (res>0) OccupiedCells[cellwidth]++;

}

}

}

fprintf(NWordOutputPrb,"%d\t%d\t%d\t", m\_TheLandscape->SupplyYearNumber() ,(int)m\_TheLandscape->SupplyDayInYear(), Counted[0] );

for (int c=0; c<4; c++) {

fprintf(NWordOutputPrb,"%d\t%d\t", totalcells[c], OccupiedCells[c]);

}

fprintf(NWordOutputPrb,"\n");

fflush(NWordOutputPrb);

}

|  |  |  |  |  |
| --- | --- | --- | --- | --- |
| virtual bool Population\_Manager::OpenTheBreedingPairsProbe | ( |  | ) |  |

inlinevirtual

Reimplemented in Skylark\_Population\_Manager.

Referenced by CreatePopulationManager().

{

return false;

}

|  |  |  |  |  |
| --- | --- | --- | --- | --- |
| virtual bool Population\_Manager::OpenTheBreedingSuccessProbe | ( |  | ) |  |

inlinevirtual

Reimplemented in Skylark\_Population\_Manager.

Referenced by CreatePopulationManager().

{

return false;

}

|  |  |  |  |  |
| --- | --- | --- | --- | --- |
| bool Population\_Manager::OpenTheCIPEGridOutputProbe | ( |  | ) |  |

open the probe

References cfg\_CIPEGridOutput\_filename, cfg\_CIPEGridOutput\_filenameB, g\_msg, CfgStr::value(), MapErrorMsg::Warn(), and WARN\_FILE.

Referenced by Skylark\_Population\_Manager::Init().

{

CIPEGridOutputPrb = fopen(cfg\_CIPEGridOutput\_filename.value(), "w" );

if ( !CIPEGridOutputPrb ) {

g\_msg->Warn( WARN\_FILE, "Population\_Manager::OpenTheCIPEGridOutputProbe(): ""Unable to open probe file",

cfg\_CIPEGridOutput\_filename.value() );

exit( 1 );

}

CIPEGridOutputPrbB = fopen(cfg\_CIPEGridOutput\_filenameB.value(), "w" );

if ( !CIPEGridOutputPrbB ) {

g\_msg->Warn( WARN\_FILE, "Population\_Manager::OpenTheCIPEGridOutputProbe(): ""Unable to open probe file",

cfg\_CIPEGridOutput\_filenameB.value() );

exit( 1 );

}

return true;

}

|  |  |  |  |  |
| --- | --- | --- | --- | --- |
| virtual bool Population\_Manager::OpenTheFledgelingProbe | ( |  | ) |  |

inlinevirtual

Reimplemented in Skylark\_Population\_Manager.

Referenced by CreatePopulationManager().

{

return false;

}

|  |  |  |  |  |
| --- | --- | --- | --- | --- |
| bool Population\_Manager::OpenTheMonthlyRipleysOutputProbe | ( |  | ) |  |

open 12 ripley output probles, one for each month

References cfg\_RipleysOutput\_filename, g\_msg, CfgStr::value(), MapErrorMsg::Warn(), and WARN\_FILE.

{

RipleysOutputPrb1 = fopen("RipleyOutput\_Jan.txt", "w" );

if ( !RipleysOutputPrb1 ) {

g\_msg->Warn( WARN\_FILE, "Population\_Manager::OpenTheRipleysOutputProbe(): ""Unable to open probe file",

cfg\_RipleysOutput\_filename.value() );

exit( 1 );

}

RipleysOutputPrb2 = fopen("RipleyOutput\_Feb.txt", "w" );

if ( !RipleysOutputPrb2 ) {

g\_msg->Warn( WARN\_FILE, "Population\_Manager::OpenTheRipleysOutputProbe(): ""Unable to open probe file",

cfg\_RipleysOutput\_filename.value() );

exit( 1 );

}

RipleysOutputPrb3 = fopen("RipleyOutput\_Mar.txt", "w" );

if ( !RipleysOutputPrb3 ) {

g\_msg->Warn( WARN\_FILE, "Population\_Manager::OpenTheRipleysOutputProbe(): ""Unable to open probe file",

cfg\_RipleysOutput\_filename.value() );

exit( 1 );

}

RipleysOutputPrb4 = fopen("RipleyOutput\_Apr.txt", "w" );

if ( !RipleysOutputPrb4 ) {

g\_msg->Warn( WARN\_FILE, "Population\_Manager::OpenTheRipleysOutputProbe(): ""Unable to open probe file",

cfg\_RipleysOutput\_filename.value() );

exit( 1 );

}

RipleysOutputPrb5 = fopen("RipleyOutput\_May.txt", "w" );

if ( !RipleysOutputPrb5 ) {

g\_msg->Warn( WARN\_FILE, "Population\_Manager::OpenTheRipleysOutputProbe(): ""Unable to open probe file",

cfg\_RipleysOutput\_filename.value() );

exit( 1 );

}

RipleysOutputPrb6 = fopen("RipleyOutput\_Jun.txt", "w" );

if ( !RipleysOutputPrb6 ) {

g\_msg->Warn( WARN\_FILE, "Population\_Manager::OpenTheRipleysOutputProbe(): ""Unable to open probe file",

cfg\_RipleysOutput\_filename.value() );

exit( 1 );

}

RipleysOutputPrb7 = fopen("RipleyOutput\_Jul.txt", "w" );

if ( !RipleysOutputPrb7 ) {

g\_msg->Warn( WARN\_FILE, "Population\_Manager::OpenTheRipleysOutputProbe(): ""Unable to open probe file",

cfg\_RipleysOutput\_filename.value() );

exit( 1 );

}

RipleysOutputPrb8 = fopen("RipleyOutput\_Aug.txt", "w" );

if ( !RipleysOutputPrb8 ) {

g\_msg->Warn( WARN\_FILE, "Population\_Manager::OpenTheRipleysOutputProbe(): ""Unable to open probe file",

cfg\_RipleysOutput\_filename.value() );

exit( 1 );

}

RipleysOutputPrb9 = fopen("RipleyOutput\_Sep.txt", "w" );

if ( !RipleysOutputPrb9 ) {

g\_msg->Warn( WARN\_FILE, "Population\_Manager::OpenTheRipleysOutputProbe(): ""Unable to open probe file",

cfg\_RipleysOutput\_filename.value() );

exit( 1 );

}

RipleysOutputPrb10 = fopen("RipleyOutput\_Oct.txt", "w" );

if ( !RipleysOutputPrb10 ) {

g\_msg->Warn( WARN\_FILE, "Population\_Manager::OpenTheRipleysOutputProbe(): ""Unable to open probe file",

cfg\_RipleysOutput\_filename.value() );

exit( 1 );

}

RipleysOutputPrb11 = fopen("RipleyOutput\_Nov.txt", "w" );

if ( !RipleysOutputPrb11 ) {

g\_msg->Warn( WARN\_FILE, "Population\_Manager::OpenTheRipleysOutputProbe(): ""Unable to open probe file",

cfg\_RipleysOutput\_filename.value() );

exit( 1 );

}

RipleysOutputPrb12 = fopen("RipleyOutput\_Dec.txt", "w" );

if ( !RipleysOutputPrb12 ) {

g\_msg->Warn( WARN\_FILE, "Population\_Manager::OpenTheRipleysOutputProbe(): ""Unable to open probe file",

cfg\_RipleysOutput\_filename.value() );

exit( 1 );

}

return true;

}

|  |  |  |  |  |
| --- | --- | --- | --- | --- |
| bool Population\_Manager::OpenTheReallyBigProbe | ( |  | ) |  |

open the probe

References cfg\_ReallyBigOutput\_filename, g\_msg, CfgStr::value(), MapErrorMsg::Warn(), and WARN\_FILE.

Referenced by Skylark\_Population\_Manager::Init().

{

ReallyBigOutputPrb = fopen(cfg\_ReallyBigOutput\_filename.value(), "w" );

if ( !ReallyBigOutputPrb ) {

g\_msg->Warn( WARN\_FILE, "Population\_Manager::OpenTheRipleysOutputProbe(): ""Unable to open probe file",

cfg\_ReallyBigOutput\_filename.value() );

exit( 1 );

}

return true;

}

|  |  |  |  |  |
| --- | --- | --- | --- | --- |
| bool Population\_Manager::OpenTheRipleysOutputProbe | ( |  | ) |  |

open the probe

References cfg\_RipleysOutput\_filename, g\_msg, CfgStr::value(), MapErrorMsg::Warn(), and WARN\_FILE.

Referenced by Skylark\_Population\_Manager::Init().

{

RipleysOutputPrb = fopen(cfg\_RipleysOutput\_filename.value(), "w" );

if ( !RipleysOutputPrb ) {

g\_msg->Warn( WARN\_FILE, "Population\_Manager::OpenTheRipleysOutputProbe(): ""Unable to open probe file",

cfg\_RipleysOutput\_filename.value() );

exit( 1 );

}

NWordOutputPrb = fopen("NWordOutputPrb.txt", "w" );

if ( !NWordOutputPrb ) {

g\_msg->Warn( WARN\_FILE, "Population\_Manager::OpenTheCIPEGridOutputProbe(): ""Unable to open NWord probe file", "" );

exit( 1 );

}

fprintf(NWordOutputPrb,"%s\t%s\t%s\t%s\t%s\t%s\t%s\t%s\t%s\t%s\t%s\t\n","Year","Day","Total no.","Cells50","Occupied50","Cells100","Occupied100","Cells200","Occupied200","Cells400","Occupied400");

return true;

}

|  |  |  |  |
| --- | --- | --- | --- |
| float Population\_Manager::Probe | ( | int | *ListIndex*, |
|  |  | probe\_data \* | *p\_TheProbe* |
|  | ) |  |  |

virtual

Default data probe. Rarely used in actuality but always available

References AnimalPosition::m\_EleType, probe\_data::m\_NoAreas, probe\_data::m\_NoEleTypes, probe\_data::m\_NoFarms, probe\_data::m\_NoVegTypes, probe\_data::m\_Rect, probe\_data::m\_RefEle, probe\_data::m\_RefFarms, probe\_data::m\_RefVeg, AnimalPosition::m\_VegType, AnimalPosition::m\_x, rectangle::m\_x1, rectangle::m\_x2, AnimalPosition::m\_y, rectangle::m\_y1, and rectangle::m\_y2.

{

// Counts through the list and goes through each area to see if the animal

// is standing there and if the farm, veg or element conditions are met

AnimalPosition Sp;

float NumberSk = 0;

// Four possibilites

// either NoVegTypes or NoElementTypes or NoFarmTypes is >0 or all==0

if ( p\_TheProbe->m\_NoFarms != 0 ) {

for ( unsigned j = 0; j < TheArray[ ListIndex ].size(); j++ ) {

Sp = TheArray[ ListIndex ] [ j ]->SupplyPosition();

unsigned Farm = TheArray[ ListIndex ] [ j ]->SupplyFarmOwnerRef();

for ( unsigned i = 0; i < p\_TheProbe->m\_NoAreas; i++ ) {

if ( ( Sp.m\_x >= p\_TheProbe->m\_Rect[ i ].m\_x1 ) && ( Sp.m\_y >= p\_TheProbe->m\_Rect[ i ].m\_y1 )

&& ( Sp.m\_x <= p\_TheProbe->m\_Rect[ i ].m\_x2 ) && ( Sp.m\_y <= p\_TheProbe->m\_Rect[ i ].m\_y2 ) )

for ( unsigned k = 0; k < p\_TheProbe->m\_NoFarms; k++ ) {

if ( p\_TheProbe->m\_RefFarms[ k ] == Farm )

NumberSk++; // it is in the square so increment number

}

}

}

} else if ( p\_TheProbe->m\_NoEleTypes != 0 ) {

for ( unsigned j = 0; j < TheArray[ ListIndex ].size(); j++ ) {

Sp = TheArray[ ListIndex ] [ j ]->SupplyPosition();

for ( unsigned i = 0; i < p\_TheProbe->m\_NoAreas; i++ ) {

if ( ( Sp.m\_x >= p\_TheProbe->m\_Rect[ i ].m\_x1 ) && ( Sp.m\_y >= p\_TheProbe->m\_Rect[ i ].m\_y1 )

&& ( Sp.m\_x <= p\_TheProbe->m\_Rect[ i ].m\_x2 ) && ( Sp.m\_y <= p\_TheProbe->m\_Rect[ i ].m\_y2 ) )

for ( unsigned k = 0; k < p\_TheProbe->m\_NoEleTypes; k++ ) {

if ( p\_TheProbe->m\_RefEle[ k ] == Sp.m\_EleType )

NumberSk++; // it is in the square so increment number

}

}

}

} else {

if ( p\_TheProbe->m\_NoVegTypes != 0 ) {

for ( unsigned j = 0; j < TheArray[ ListIndex ].size(); j++ ) {

Sp = TheArray[ ListIndex ] [ j ]->SupplyPosition();

for ( unsigned i = 0; i < p\_TheProbe->m\_NoAreas; i++ ) {

if ( ( Sp.m\_x >= p\_TheProbe->m\_Rect[ i ].m\_x1 ) && ( Sp.m\_y >= p\_TheProbe->m\_Rect[ i ].m\_y1 )

&& ( Sp.m\_x <= p\_TheProbe->m\_Rect[ i ].m\_x2 ) && ( Sp.m\_y <= p\_TheProbe->m\_Rect[ i ].m\_y2 ) ) {

for ( unsigned k = 0; k < p\_TheProbe->m\_NoVegTypes; k++ ) {

if ( p\_TheProbe->m\_RefVeg[ k ] == Sp.m\_VegType )

NumberSk++; // it is in the square so increment number

}

}

}

}

} else // both must be zero

{

unsigned sz = (unsigned) TheArray[ ListIndex ].size();

// It is worth checking whether we have a total dump of all individuals

// if so don't bother with asking each where it is

if (p\_TheProbe->m\_NoAreas==1) {

if ((p\_TheProbe->m\_Rect[0].m\_x1==0) && (p\_TheProbe->m\_Rect[0].m\_y1==0) && (p\_TheProbe->m\_Rect[0].m\_x2==(unsigned)SimW) &&

(p\_TheProbe->m\_Rect[0].m\_y2==(unsigned)SimH)) {

return (float) sz;

}

}

// Asking for a subset - need to test them all

for ( unsigned j = 0; j < sz; j++ ) {

Sp = TheArray[ ListIndex ] [ j ]->SupplyPosition();

for ( unsigned i = 0; i < p\_TheProbe->m\_NoAreas; i++ ) {

if ( ( Sp.m\_x >= p\_TheProbe->m\_Rect[ i ].m\_x1 ) && ( Sp.m\_y >= p\_TheProbe->m\_Rect[ i ].m\_y1 )

&& ( Sp.m\_x <= p\_TheProbe->m\_Rect[ i ].m\_x2 ) && ( Sp.m\_y <= p\_TheProbe->m\_Rect[ i ].m\_y2 ) )

NumberSk++; // it is in the square so increment number

}

}

}

}

return NumberSk;

}

|  |  |  |  |
| --- | --- | --- | --- |
| int Population\_Manager::ProbeFileInput | ( | char \* | *p\_Filename*, |
|  |  | int | *p\_ProbeNo* |
|  | ) |  |  |

Default probe file input

Referenced by GetProbeInput\_ini().

{

FILE \* PFile;

int data = 0;

int data2 = 0;

char S[ 255 ];

PFile = fopen(p\_Filename, "r" );

if ( !PFile ) {

m\_TheLandscape->Warn( "Population Manager - cannot open Probe File ", p\_Filename );

exit( 0 );

}

fgets( S, 255, PFile ); // dummy line

fgets( S, 255, PFile ); // dummy line

fscanf( PFile, "%d\n", & data ); // Reporting interval

TheProbe[ p\_ProbeNo ]->m\_ReportInterval = data;

fgets( S, 255, PFile ); // dummy line

fscanf( PFile, "%d\n", & data ); // Write to file

if ( data == 0 ) TheProbe[ p\_ProbeNo ]->m\_FileRecord = false; else

TheProbe[ p\_ProbeNo ]->m\_FileRecord = true;

fgets( S, 255, PFile ); // dummy line

for ( int i = 0; i < 10; i++ ) {

fscanf( PFile, "%d", & data );

if ( data > 0 ) TheProbe[ p\_ProbeNo ]->m\_TargetTypes[ i ] = true; else

TheProbe[ p\_ProbeNo ]->m\_TargetTypes[ i ] = false;

}

fgets( S, 255, PFile ); // dummy line

fgets( S, 255, PFile ); // dummy line

fscanf( PFile, "%d", & data );

TheProbe[ p\_ProbeNo ]->m\_NoAreas = data;

fgets( S, 255, PFile ); // dummy line

fgets( S, 255, PFile ); // dummy line

fscanf( PFile, "%d", & data2 ); // No References areas

fgets( S, 255, PFile ); // dummy line

fgets( S, 255, PFile ); // dummy line

fscanf( PFile, "%d", & data ); // Type reference for probe

if ( data == 1 ) TheProbe[ p\_ProbeNo ]->m\_NoEleTypes = data2; else

TheProbe[ p\_ProbeNo ]->m\_NoEleTypes = 0;

if ( data == 2 ) TheProbe[ p\_ProbeNo ]->m\_NoVegTypes = data2; else

TheProbe[ p\_ProbeNo ]->m\_NoVegTypes = 0;

if ( data == 3 ) TheProbe[ p\_ProbeNo ]->m\_NoFarms = data2; else

TheProbe[ p\_ProbeNo ]->m\_NoFarms = 0;

fgets( S, 255, PFile ); // dummy line

fgets( S, 255, PFile ); // dummy line

// Now read in the areas data

for ( int i = 0; i < 10; i++ ) {

fscanf( PFile, "%d", & data );

TheProbe[ p\_ProbeNo ]->m\_Rect[ i ].m\_x1 = data;

fscanf( PFile, "%d", & data );

TheProbe[ p\_ProbeNo ]->m\_Rect[ i ].m\_y1 = data;

fscanf( PFile, "%d", & data );

TheProbe[ p\_ProbeNo ]->m\_Rect[ i ].m\_x2 = data;

fscanf( PFile, "%d", & data );

TheProbe[ p\_ProbeNo ]->m\_Rect[ i ].m\_y2 = data;

}

fgets( S, 255, PFile ); // dummy line

fgets( S, 255, PFile ); // dummy line

if ( TheProbe[ p\_ProbeNo ]->m\_NoVegTypes > 0 ) {

for ( int i = 0; i < 25; i++ ) {

fscanf( PFile, "%d", & data );

if ( data != 999 )

TheProbe[ p\_ProbeNo ]->m\_RefVeg[ i ] = m\_TheLandscape->TranslateVegTypes( data );

}

} else if ( TheProbe[ p\_ProbeNo ]->m\_NoFarms > 0 ) {

for ( int i = 0; i < 25; i++ ) {

fscanf( PFile, "%d", & data );

if ( data != 999 )

TheProbe[ p\_ProbeNo ]->m\_RefFarms[ i ] = data;

}

} else {

for ( int i = 0; i < 25; i++ ) {

fscanf( PFile, "%d", & data );

if ( data != 999 ) TheProbe[ p\_ProbeNo ]->m\_RefEle[ i ] = m\_TheLandscape->TranslateEleTypes( data );

}

}

fclose( PFile );

return data2; // number of data references

}

|  |  |  |  |  |  |
| --- | --- | --- | --- | --- | --- |
| char \* Population\_Manager::ProbeReport | ( | int | *a\_time* | ) |  |

References BeginningOfMonth(), and g\_str.

{

int No;

char str[100]; // 100 out to be enough!!

strcpy(g\_str,"");

bool found = false;

for ( int ProbeNo = 0; ProbeNo < m\_NoProbes; ProbeNo++ ) {

No = 0;

// See if we need to record/update this one

// if time/months/years ==0 or every time

if ( ( TheProbe[ ProbeNo ]->m\_ReportInterval == 3 )

|| ( ( TheProbe[ ProbeNo ]->m\_ReportInterval == 2 ) && ( BeginningOfMonth() ) )

|| ( ( TheProbe[ ProbeNo ]->m\_ReportInterval == 1 ) && ( Time % 365 == 0 ) ) ) {

// Goes through each area and sends a value to OutputForm

unsigned Index = SupplyListIndexSize();

for ( unsigned listindex = 0; listindex < Index; listindex++ ) {

if ( TheProbe[ ProbeNo ]->m\_TargetTypes[ listindex ] )

No += (int) Probe( listindex, TheProbe[ ProbeNo ] );

}

TheProbe[ ProbeNo ]->FileOutput( No, Time, ProbeNo );

sprintf(str," %d ", No );

strcat(g\_str,str);

found = true;

}

}

return g\_str;

}

|  |  |  |  |  |  |
| --- | --- | --- | --- | --- | --- |
| char \* Population\_Manager::ProbeReportTimed | ( | int | *a\_time* | ) |  |

References g\_str.

{

int No;

char str[100]; // 100 ought to be enough!!

strcpy(g\_str,"");

for ( int ProbeNo = 0; ProbeNo < m\_NoProbes; ProbeNo++ ) {

No = 0;

unsigned Index = SupplyListIndexSize();

for ( unsigned listindex = 0; listindex < Index; listindex++ ) {

if ( TheProbe[ ProbeNo ]->m\_TargetTypes[ listindex ] ) No += (int) Probe( listindex, TheProbe[ ProbeNo ] );

}

TheProbe[ ProbeNo ]->FileOutput( No, Time, ProbeNo );

sprintf(str," %d ", No );

strcat(g\_str,str);

}

return g\_str;

}

|  |  |  |  |  |  |
| --- | --- | --- | --- | --- | --- |
| void Population\_Manager::Run | ( | int | *NoTSteps* | ) |  |

virtual

This is the main scheduling method for the population manager.   
Note the structure of Shuffle\_or\_Sort(), DoFirst(), BeginStep, DoBefore(), Step looping until all are finished, DoAfter(), DoAlmostLast(), EndStep, DoLast().

Can do multiple time-steps here inside one landscape time-step (a day). This is used in the roe deer model to provide 10 minute behavioural time-steps.

References cfg\_CatastropheEventStartYear, cfg\_CIPEGridOutput\_day, cfg\_CIPEGridOutput\_Interval, cfg\_CIPEGridOutput\_used, cfg\_ReallyBigOutput\_day1, cfg\_ReallyBigOutput\_day2, cfg\_ReallyBigOutput\_day3, cfg\_ReallyBigOutput\_day4, cfg\_ReallyBigOutput\_interval, cfg\_ReallyBigOutput\_used, cfg\_ReallyBigOutputFirstYear, cfg\_RipleysOutput\_day, cfg\_RipleysOutput\_interval, cfg\_RipleysOutput\_used, cfg\_RipleysOutputFirstYear, cfg\_RipleysOutputMonthly\_used, CfgInt::value(), and CfgBool::value().

Referenced by RunTheSim().

{

// begin step actions ...

// set all stepdone to false.... is this really necessary??

unsigned size2;

unsigned size1 = (unsigned) TheArray.size();

for ( unsigned listindex = 0; listindex < size1; listindex++ ) {

size2 = (unsigned) TheArray[ listindex ].size();

for ( unsigned j = 0; j < size2; j++ ) {

TheArray[ listindex ] [ j ]->StepDone = false;

}

}

for ( int TSteps = 0; TSteps < NoTSteps; TSteps++ ) {

for ( unsigned listindex = 0; listindex < size1; listindex++ ) {

// Call the Shuffle/Sort procedures

Shuffle\_or\_Sort( listindex );

}

// Need to check if Ripleys Statistic needs to be saved

if ( cfg\_RipleysOutput\_used.value() ) {

int Year = m\_TheLandscape->SupplyYearNumber();

if (Year>=cfg\_RipleysOutputFirstYear.value()) {

if ( Year % cfg\_RipleysOutput\_interval.value() == 0 ) {

int day = m\_TheLandscape->SupplyDayInYear();

if ( cfg\_RipleysOutput\_day.value() == day ) {

// Do the Ripley Probe

TheRipleysOutputProbe( RipleysOutputPrb );

TheNWordOutputProbe(); // These two go hand in hand.

}

}

}

}

// Need to check if Monthly Ripleys Statistic needs to be saved

if ( cfg\_RipleysOutputMonthly\_used.value() ) {

if (m\_TheLandscape->SupplyDayInMonth()==1) {

int Year = m\_TheLandscape->SupplyYearNumber();

if (Year>=cfg\_RipleysOutputFirstYear.value()) {

if ( Year % cfg\_RipleysOutput\_interval.value() == 0 ) {

int month = m\_TheLandscape->SupplyMonth();

// Do the Ripley Probe

switch (month) {

case 1: TheRipleysOutputProbe( RipleysOutputPrb1 );

break;

case 2: TheRipleysOutputProbe( RipleysOutputPrb2 );

break;

case 3: TheRipleysOutputProbe( RipleysOutputPrb3 );

break;

case 4: TheRipleysOutputProbe( RipleysOutputPrb4 );

break;

case 5: TheRipleysOutputProbe( RipleysOutputPrb5 );

break;

case 6: TheRipleysOutputProbe( RipleysOutputPrb6 );

break;

case 7: TheRipleysOutputProbe( RipleysOutputPrb7 );

break;

case 8: TheRipleysOutputProbe( RipleysOutputPrb8 );

break;

case 9: TheRipleysOutputProbe( RipleysOutputPrb9 );

break;

case 10: TheRipleysOutputProbe( RipleysOutputPrb10 );

break;

case 11: TheRipleysOutputProbe( RipleysOutputPrb11 );

break;

case 12: TheRipleysOutputProbe( RipleysOutputPrb12 );

break;

}

}

}

}

}

// Need to check if Really Big Probe needs to be saved

if ( cfg\_ReallyBigOutput\_used.value() ) {

int Year = m\_TheLandscape->SupplyYearNumber();

if (Year>=cfg\_ReallyBigOutputFirstYear.value()) {

if ( Year % cfg\_ReallyBigOutput\_interval.value() == 0 ) {

int day = m\_TheLandscape->SupplyDayInYear();

if (( cfg\_ReallyBigOutput\_day1.value() == day )|| ( cfg\_ReallyBigOutput\_day2.value() == day )|| ( cfg\_ReallyBigOutput\_day3.value() == day )|| ( cfg\_ReallyBigOutput\_day4.value() == day )||( cfg\_ReallyBigOutput\_day1.value() == -1 ))

{

// Do the Ripley Probe

TheReallyBigOutputProbe();

}

}

}

}

// Need to check if CIPEGrid Statistic needs to be saved

if ( cfg\_CIPEGridOutput\_used.value() ) {

int Year = m\_TheLandscape->SupplyYearNumber();

if ( Year % cfg\_CIPEGridOutput\_Interval.value() == 0 ) {

int day = m\_TheLandscape->SupplyDayInYear();

if ( cfg\_CIPEGridOutput\_day.value() == day ) {

// Do the Ripley Probe

TheCIPEGridOutputProbe();

}

}

}

int yr=m\_TheLandscape->SupplyYearNumber();

if ( yr > cfg\_CatastropheEventStartYear.value() ) {

if (m\_catastrophestartyear==-1) m\_catastrophestartyear=yr;

Catastrophe(); // This method must be overidden in descendent classes

}

DoFirst();

// call the begin-step-method of all objects

for ( unsigned listindex = 0; listindex < size1; listindex++ ) {

size2 = (unsigned) TheArray[ listindex ].size();

for ( unsigned j = 0; j < size2; j++ )

TheArray[ listindex ] [ j ]->BeginStep();

}

DoBefore();

// call the step-method of all objects

do {

for ( unsigned listindex = 0; listindex < size1; listindex++ ) {

//DEBUG

//LOG("SecondLog.txt");

size2 = (unsigned) TheArray[ listindex ].size();

for ( unsigned j = 0; j < size2; j++ ) {

TheArray[ listindex ] [ j ]->Step();

}

} // for listindex

} while ( !StepFinished() );

DoAfter();

// call the end-step-method of all objects

for ( unsigned listindex = 0; listindex < size1; listindex++ ) {

size2 = (int) TheArray[ listindex ].size();

for ( unsigned j = 0; j < size2; j++ ) {

TheArray[ listindex ] [ j ]->EndStep();

}

}

// ----------------

// end of this step actions

// For each animal list

DoAlmostLast();

for ( unsigned listindex = 0; listindex < size1; listindex++ ) {

// Must check each object in the list for CurrentStateNo==-1

SortStateR(listindex);

vector<TAnimal\*>::reverse\_iterator ritE=TheArray[listindex].rend();

vector<TAnimal\*>::reverse\_iterator ritB=TheArray[listindex].rbegin();

while (ritB!=ritE) {

if ((\*ritB)->CurrentStateNo<0) delete (\*ritB++); else break;

}

TheArray[listindex].erase((ritB).base(),TheArray[listindex].end());

}

/\*/

for ( unsigned listindex = 0; listindex < size1; listindex++ ) {

// Must check each object in the list for CurrentStateNo==-1

int TAend = (int) TheArray[ listindex ].size() - 1;

for ( int j = TAend; j >= 0; j-- ) // Search backwards is more efficicent

{

if ( TheArray[ listindex ] [ j ]->CurrentStateNo == -1 ) // code for kill it

{

delete TheArray[ listindex ] [ j ];

TheArray[ listindex ].erase( TheArray[ listindex ].begin() + j );

}

}

}

\*/

DoLast();

} // End of time step loop

}

|  |  |  |  |  |  |
| --- | --- | --- | --- | --- | --- |
| void Population\_Manager::SetNoProbes | ( | int | *a\_pn* | ) |  |

inline

References m\_NoProbes.

Referenced by CreatePopulationManager().

{ m\_NoProbes=a\_pn; }

|  |  |  |  |  |  |
| --- | --- | --- | --- | --- | --- |
| void Population\_Manager::Shuffle | ( | unsigned | *Type* | ) |  |

protected

Run once through the list swapping randomly chosen elements

References random().

{

unsigned s = (unsigned) TheArray[ Type ].size();

for ( unsigned i = 0; i < s; i++ ) {

TAnimal \* temp;

unsigned a = random( s );

unsigned b = random( s );

temp = TheArray[ Type ] [ a ];

TheArray[ Type ] [ a ] = TheArray[ Type ] [ b ];

TheArray[ Type ] [ b ] = temp;

}

}

|  |  |  |  |  |  |
| --- | --- | --- | --- | --- | --- |
| void Population\_Manager::Shuffle\_or\_Sort | ( | unsigned | *Type* | ) |  |

protected

This method is used to determine whether the array of animals should be shuffled or sorted.   
To do nothing simply avoid calling this at the beginning of the time-step

{

switch ( BeforeStepActions[ Type ] ) {

case 0:

Shuffle( Type );

break;

case 1:

SortX( Type );

break;

case 2:

SortY( Type );

break;

case 3:

SortXIndex( Type );

break;

default:

m\_TheLandscape->Warn( "Population\_Manager::Shuffle\_or\_Sort- BeforeStepAction Unknown", NULL );

exit( 1 );

}

}

|  |  |  |  |  |  |
| --- | --- | --- | --- | --- | --- |
| void Population\_Manager::SortState | ( | unsigned | *Type* | ) |  |

protected

Sort TheArray w.r.t. the current state attribute

{

sort( TheArray[ Type ].begin(), TheArray[ Type ].end(), CompareState() );

}

|  |  |  |  |  |  |
| --- | --- | --- | --- | --- | --- |
| void Population\_Manager::SortStateR | ( | unsigned | *Type* | ) |  |

protected

Sort TheArray w.r.t. the current state attribute in reverse order

{

sort( TheArray[ Type ].begin(), TheArray[ Type ].end(), CompareStateR() );

}

|  |  |  |  |  |  |
| --- | --- | --- | --- | --- | --- |
| void Population\_Manager::SortX | ( | unsigned | *Type* | ) |  |

protected

Sort TheArray w.r.t. the m\_Location\_x attribute

{

sort( TheArray[ Type ].begin(), TheArray[ Type ].end(), CompareX() );

}

|  |  |  |  |  |  |
| --- | --- | --- | --- | --- | --- |
| void Population\_Manager::SortXIndex | ( | unsigned | *Type* | ) |  |

protected

Sort TheArray w.r.t. the m\_Location\_x attribute, and make an indexing array

{

sort( TheArray[ Type ].begin(), TheArray[ Type ].end(), CompareX() );

unsigned s = (unsigned) TheArray[ Type ].size();

// Now make the index array to X;

int counter = 0;

int x;

// for each individual

for ( unsigned i = 0; i < s; i++ ) {

// Get Next co-ordiated

x = TheArray[ Type ] [ i ]->Supply\_m\_Location\_x();

// fill gaps up to x

while ( counter < x ) IndexArrayX[ Type ] [ counter++ ] = -1;

if ( x == counter ) {

IndexArrayX[ Type ] [ counter++ ] = i;

}

}

// Fill up the rest of the array with -1;

for ( int c = counter; c < 10000; c++ ) {

IndexArrayX[ Type ] [ c ] = -1;

}

}

|  |  |  |  |  |  |
| --- | --- | --- | --- | --- | --- |
| void Population\_Manager::SortY | ( | unsigned | *Type* | ) |  |

protected

Sort TheArray w.r.t. the m\_Location\_y attribute

{

sort( TheArray[ Type ].begin(), TheArray[ Type ].end(), CompareY() );

}

|  |  |  |  |
| --- | --- | --- | --- |
| char \* Population\_Manager::SpeciesSpecificReporting | ( | int | *a\_species*, |
|  |  | int | *a\_time* |
|  | ) |  |  |

This method handles species specific outputs. This is one place to do it. More commonly this is done in descendent classes

References g\_str, ImpactProbeReport(), and ProbeReport().

Referenced by RunTheSim().

{

strcpy(g\_str,"");

// Vole Model

if ( a\_species == 1 ) {

/\*

// The next lines are obselete code from genetic simulations

float MeanNoAlleles;

float MeanHO;

float MeanHE;

unsigned size;

if ( m\_time % ( 365 \* 24 \* 60 ) == 0 ) // End of year

{

// empty the GenStruc data

for ( int ProbeNo = 0; ProbeNo < ( int )m\_NoProbes; ProbeNo++ ) {

fprintf( m\_GeneticsFile, "%d ", m\_time );

int TotalSize = 0;

m\_AManager->AlFreq->Flush();

for ( unsigned listindex = 0; listindex <= 1; listindex++ ) {

size = 0;

m\_AManager->TheGeneticProbe( listindex, ProbeNo, size );

TotalSize += size;

}

if ( TotalSize > 0 ) {

m\_AManager->AlFreq->CalcAF();

m\_AManager->AlFreq->CalcNoAlleles();

m\_AManager->AlFreq->CalcHE();

m\_AManager->AlFreq->CalcHO( TotalSize );

}

// Get the means

// For N alleles

int NA = 0;

float NHO = 0;

float NHE = 0;

for ( int l = 0; l < 16; l++ ) {

NA += m\_AManager->AlFreq->SupplyNoAlleles( l );

}

MeanNoAlleles = ( float )NA / 16.0;

for ( int l = 0; l < 16; l++ ) {

NHO += m\_AManager->AlFreq->SupplyHO( l );

}

MeanHO = NHO / 16.0;

for ( int l = 0; l < 16; l++ ) {

NHE += m\_AManager->AlFreq->SupplyHE( l );

}

MeanHE = NHE / 16.0;

// Do some output

// Output is to two files, the genetics file and the AllelerequencyFile

fprintf( m\_GeneticsFile, "%2.2f ", MeanNoAlleles );

fprintf( m\_GeneticsFile, "%1.3f ", MeanHO );

fprintf( m\_GeneticsFile, "%1.3f ", MeanHE );

for ( int l = 0; l < 16; l++ ) {

fprintf( m\_GeneticsFile, "%04i ", m\_AManager->AlFreq->SupplyNoAlleles( l ) );

}

for ( int l = 0; l < 16; l++ ) {

fprintf( m\_GeneticsFile, "%1.3f ", m\_AManager->AlFreq->SupplyHO( l ) );

}

for ( int l = 0; l < 16; l++ ) {

fprintf( m\_GeneticsFile, "%1.3f ", m\_AManager->AlFreq->SupplyHE( l ) );

}

fprintf( m\_GeneticsFile, "\n" );

fprintf( m\_AlleleFreqsFile, "%d ", m\_time );

for ( int l = 0; l < 16; l++ ) {

for ( int al = 0; al < 4; al++ ) {

fprintf( m\_AlleleFreqsFile, "%1.3f ", m\_AManager->AlFreq->SupplyAF( l, al ) );

}

}

fprintf( m\_AlleleFreqsFile, "\n" );

}

// make sure progress reaches the disk

fflush( m\_GeneticsFile );

fflush( m\_AlleleFreqsFile );

}

if ( cfg\_UseEasyPop.value()) {

int targettime=a\_time % ( 365 \* 100 );

if ( ( targettime == 59 ) ||(targettime == 181)||(targettime ==304)||(targettime ==59+365)||(targettime ==181+365)||(targettime ==304+365)) {

for ( unsigned listindex = 0; listindex <= 1; listindex++ ) {

GeneticsResultsOutput( m\_EasyPopRes, listindex);

}

fflush( m\_EasyPopRes );

}

}

\*/

/\* Three times a year reporting

if ( m\_time % 365 == 60 ) ProbeReportNow( m\_time );

if ( m\_time % 365 == 152 ) ProbeReportNow( m\_time );

if ( m\_time % 365 == 273 ) ProbeReportNow( m\_time ); \*/

ProbeReport( a\_time );

#ifdef \_\_SpecificPesticideEffectsVinclozolinLike\_\_

ImpactProbeReport( a\_time );

#endif

#ifdef \_\_WithinOrchardPesticideSim\_\_

ImpactProbeReport( a\_time );

#endif

}

// Skylark Model

else if ( a\_species == 0 ) {

int No;

int a\_day = a\_time % 365;

if ( a\_time % 365 == 364 )

{

//Write the Breeding Attempts Probe

int BreedingFemales, YoungOfTheYear, TotalPop, TotalFemales, TotalMales, BreedingAttempts;

int No = TheBreedingSuccessProbe( BreedingFemales, YoungOfTheYear, TotalPop, TotalFemales, TotalMales, BreedingAttempts );

float bs = 0;

if ( BreedingFemales > 0 ) {

bs = No / ( float )BreedingFemales; //bs = successful breeding attempt per attempting to breed female

}

BreedingSuccessProbeOutput( bs, BreedingFemales, YoungOfTheYear, TotalPop,

TotalFemales, TotalMales, a\_time, BreedingAttempts );

}

if ( a\_day == 152 ) {

// Need to fill in the landscape from 1st June, it will change before

// the count of fledgelings is needed

m\_TheLandscape->FillVegAreaData();

}

if ( a\_day == 197 ) {

for ( int ProbeNo = 0; ProbeNo < m\_NoProbes; ProbeNo++ ) {

No = TheFledgelingProbe();

// Do some output

FledgelingProbeOutput( No, a\_time );

}

}

#ifdef \_\_SKPOM

// This is clunky but for purposes of validation we want probes throughout the year

// First get the breeding pairs data.

BreedingPairsOutput(a\_day);

#else

ProbeReport( a\_time );

#endif

}

else return ProbeReport( a\_time );

return g\_str;

}

|  |  |  |  |  |  |
| --- | --- | --- | --- | --- | --- |
| bool Population\_Manager::StepFinished | ( | void |  | ) |  |

protectedvirtual

Returns true if and only if all objects have finished the current step

{

for ( unsigned listindex = 0; listindex < TheArray.size(); listindex++ ) {

for ( unsigned j = 0; j < TheArray[ listindex ].size(); j++ ) {

if ( TheArray[ listindex ] [ j ]->StepDone == false ) {

return false;

}

}

}

return true;

}

|  |  |  |  |  |  |
| --- | --- | --- | --- | --- | --- |
| virtual int Population\_Manager::SupplyCovPosx | ( | int |  | ) |  |

inlinevirtual

{

return 0;

}

|  |  |  |  |  |  |
| --- | --- | --- | --- | --- | --- |
| virtual int Population\_Manager::SupplyCovPosy | ( | int |  | ) |  |

inlinevirtual

{

return 0;

}

|  |  |  |  |  |
| --- | --- | --- | --- | --- |
| unsigned Population\_Manager::SupplyListIndexSize | ( |  | ) |  |

inline

References TheArray.

Referenced by Skylark\_Population\_Manager::ProbeReportPOM().

{

return (unsigned)TheArray.size();

}

|  |  |  |  |  |  |
| --- | --- | --- | --- | --- | --- |
| const char\* Population\_Manager::SupplyListName | ( | int | *i* | ) |  |

inline

References m\_ListNames.

{

return m\_ListNames[ i ];

}

|  |  |  |  |  |
| --- | --- | --- | --- | --- |
| int Population\_Manager::SupplyListNameLength | ( |  | ) |  |

inline

References m\_ListNameLength.

{

return m\_ListNameLength;

}

|  |  |  |  |  |  |
| --- | --- | --- | --- | --- | --- |
| unsigned Population\_Manager::SupplyListSize | ( | unsigned | *listindex* | ) |  |

inline

References TheArray.

{

return (unsigned) TheArray[ listindex ].size();

}

|  |  |  |  |
| --- | --- | --- | --- |
| virtual void Population\_Manager::SupplyLocXY | ( | unsigned | *listindex*, |
|  |  | unsigned | *j*, |
|  |  | int & | *x*, |
|  |  | int & | *y* |
|  | ) |  |  |

inlinevirtual

References TheArray.

{

x = TheArray[ listindex ] [ j ]->Supply\_m\_Location\_x();

y = TheArray[ listindex ] [ j ]->Supply\_m\_Location\_y();

}

|  |  |  |  |  |  |
| --- | --- | --- | --- | --- | --- |
| virtual int Population\_Manager::SupplyPegPosx | ( | int |  | ) |  |

inlinevirtual

{

return 0;

}

|  |  |  |  |  |  |
| --- | --- | --- | --- | --- | --- |
| virtual int Population\_Manager::SupplyPegPosy | ( | int |  | ) |  |

inlinevirtual

{

return 0;

}

|  |  |  |  |  |
| --- | --- | --- | --- | --- |
| int Population\_Manager::SupplySimH | ( |  | ) |  |

inline

References SimH.

Referenced by Skylark\_Female::st\_Floating(), Skylark\_Male::st\_Floating(), and Skylark\_Female::st\_Laying().

{

return SimH;

}

|  |  |  |  |  |
| --- | --- | --- | --- | --- |
| int Population\_Manager::SupplySimW | ( |  | ) |  |

inline

References SimW.

Referenced by Skylark\_Female::st\_Floating(), Skylark\_Male::st\_Floating(), and Skylark\_Female::st\_Laying().

{

return SimW;

}

|  |  |  |  |
| --- | --- | --- | --- |
| int Population\_Manager::SupplyState | ( | unsigned | *listindex*, |
|  |  | unsigned | *j* |
|  | ) |  |  |

inline

References TheArray.

{

return TheArray[ listindex ] [ j ]->WhatState();

}

|  |  |  |  |  |
| --- | --- | --- | --- | --- |
| IntArray100\* Population\_Manager::SupplyStateList | ( |  | ) |  |

inline

References StateList.

{

return & StateList;

}

|  |  |  |  |  |  |
| --- | --- | --- | --- | --- | --- |
| const char\* Population\_Manager::SupplyStateNames | ( | int | *i* | ) |  |

inline

References StateNames.

Referenced by Skylark\_Female::Step(), and Skylark\_Male::Step().

{

return StateNames[ i ];

}

|  |  |  |  |  |
| --- | --- | --- | --- | --- |
| unsigned Population\_Manager::SupplyStateNamesLength | ( |  | ) |  |

inline

References StateNamesLength.

{

return StateNamesLength;

}

|  |  |  |  |  |
| --- | --- | --- | --- | --- |
| int Population\_Manager::SupplyStepSize | ( |  | ) |  |

inline

References m\_StepSize.

{

return m\_StepSize;

}

|  |  |  |  |  |  |
| --- | --- | --- | --- | --- | --- |
| virtual int Population\_Manager::TheBreedingFemalesProbe | ( | int |  | ) |  |

inlinevirtual

Reimplemented in Skylark\_Population\_Manager.

{

return 0;

}

|  |  |  |  |
| --- | --- | --- | --- |
| virtual int Population\_Manager::TheBreedingSuccessProbe | ( | int & | , |
|  |  | int & | , |
|  |  | int & | , |
|  |  | int & | , |
|  |  | int & | , |
|  |  | int & |  |
|  | ) |  |  |

inlinevirtual

Reimplemented in Skylark\_Population\_Manager.

{

return 0;

}

|  |  |  |  |  |
| --- | --- | --- | --- | --- |
| void Population\_Manager::TheCIPEGridOutputProbe | ( |  | ) |  |

virtual

This method MUST be overridden in descendent classes, if you want it to work

Reimplemented in Skylark\_Population\_Manager.

{

}

|  |  |  |  |  |
| --- | --- | --- | --- | --- |
| virtual int Population\_Manager::TheFledgelingProbe | ( |  | ) |  |

inlinevirtual

Reimplemented in Skylark\_Population\_Manager.

{

return 0;

}

|  |  |  |  |
| --- | --- | --- | --- |
| virtual void Population\_Manager::TheGeneticProbe | ( | unsigned | , |
|  |  | int | , |
|  |  | unsigned & |  |
|  | ) |  |  |

inlinevirtual

{

}

|  |  |  |  |  |
| --- | --- | --- | --- | --- |
| virtual void Population\_Manager::TheNWordOutputProbe | ( |  | ) |  |

inlinevirtual

Reimplemented in Skylark\_Population\_Manager.

{};

|  |  |  |  |  |
| --- | --- | --- | --- | --- |
| void Population\_Manager::TheReallyBigOutputProbe | ( |  | ) |  |

virtual

This method must be overridden in descendent classes

{

}

|  |  |  |  |  |  |
| --- | --- | --- | --- | --- | --- |
| void Population\_Manager::TheRipleysOutputProbe | ( | FILE \* | *a\_prb* | ) |  |

virtual

This method must be overridden in descendent classes

Reimplemented in Skylark\_Population\_Manager.

{

}

---

## Member Data Documentation

AlleleFreq\* Population\_Manager::AlFreq

unsigned Population\_Manager::BeforeStepActions[10]

protected

Referenced by Skylark\_Population\_Manager::Init().

FILE\* Population\_Manager::CIPEGridOutputPrb

protected

Referenced by Skylark\_Population\_Manager::TheCIPEGridOutputProbe().

FILE\* Population\_Manager::CIPEGridOutputPrbB

protected

unsigned Population\_Manager::Counts[10][100]

int Population\_Manager::IndexArrayX[5][10000]

long int Population\_Manager::lamdagrid[2][257][257]

protected

Referenced by LamdaBirth(), LamdaClear(), and LamdaDeath().

FILE\* Population\_Manager::m\_AlleleFreqsFile

protected

int Population\_Manager::m\_catastrophestartyear

protected

Referenced by Skylark\_Population\_Manager::Catastrophe().

int\* Population\_Manager::m\_cgridcount

protected

Referenced by Skylark\_Population\_Manager::TheCIPEGridOutputProbe().

int Population\_Manager::m\_cgridcountwidth

protected

Referenced by Skylark\_Population\_Manager::TheCIPEGridOutputProbe().

int Population\_Manager::m\_cipegridsize

protected

Referenced by Skylark\_Population\_Manager::TheCIPEGridOutputProbe().

FILE\* Population\_Manager::m\_EasyPopRes

protected

FILE\* Population\_Manager::m\_GeneticsFile

protected

int\* Population\_Manager::m\_gridcount[4]

protected

Referenced by Skylark\_Population\_Manager::TheNWordOutputProbe().

int Population\_Manager::m\_gridcountsize[4]

protected

Referenced by Skylark\_Population\_Manager::TheNWordOutputProbe().

int Population\_Manager::m\_gridcountwidth[4]

protected

Referenced by Skylark\_Population\_Manager::TheNWordOutputProbe().

unsigned Population\_Manager::m\_ListNameLength

protected

Referenced by Skylark\_Population\_Manager::Init(), and SupplyListNameLength().

const char\* Population\_Manager::m\_ListNames[10]

protected

Referenced by Skylark\_Population\_Manager::Init(), and SupplyListName().

MyDialog\* Population\_Manager::m\_MainForm

int Population\_Manager::m\_NoProbes

protected

Referenced by Skylark\_Population\_Manager::ProbeReportPOM(), and SetNoProbes().

char Population\_Manager::m\_SimulationName[255]

Referenced by Skylark\_Population\_Manager::Init().

int Population\_Manager::m\_StepSize

protected

Referenced by SupplyStepSize().

Landscape\* Population\_Manager::m\_TheLandscape

Referenced by Skylark\_Population\_Manager::Catastrophe(), Skylark\_Population\_Manager::DoFirst(), Skylark\_Population\_Manager::FledgelingProbeOutput(), Skylark\_Population\_Manager::Init(), Skylark\_Population\_Manager::OpenTheFledgelingProbe(), and Skylark\_Population\_Manager::TheRipleysOutputProbe().

FILE\* Population\_Manager::NWordOutputPrb

protected

bool Population\_Manager::ProbesSet

FILE\* Population\_Manager::ReallyBigOutputPrb

protected

Referenced by Skylark\_Population\_Manager::Init().

FILE\* Population\_Manager::RipleysOutputPrb

protected

FILE\* Population\_Manager::RipleysOutputPrb1

protected

FILE\* Population\_Manager::RipleysOutputPrb10

protected

FILE\* Population\_Manager::RipleysOutputPrb11

protected

FILE\* Population\_Manager::RipleysOutputPrb12

protected

FILE\* Population\_Manager::RipleysOutputPrb2

protected

FILE\* Population\_Manager::RipleysOutputPrb3

protected

FILE\* Population\_Manager::RipleysOutputPrb4

protected

FILE\* Population\_Manager::RipleysOutputPrb5

protected

FILE\* Population\_Manager::RipleysOutputPrb6

protected

FILE\* Population\_Manager::RipleysOutputPrb7

protected

FILE\* Population\_Manager::RipleysOutputPrb8

protected

FILE\* Population\_Manager::RipleysOutputPrb9

protected

int Population\_Manager::SimH

Referenced by Skylark\_Male::EstablishingATerritory(), Skylark\_Female::EstablishTerritory(), and SupplySimH().

unsigned Population\_Manager::SimHH

int Population\_Manager::SimW

Referenced by Skylark\_Male::EstablishingATerritory(), Skylark\_Female::EstablishTerritory(), and SupplySimW().

unsigned Population\_Manager::SimWH

IntArray100 Population\_Manager::StateList

protected

Referenced by SupplyStateList().

const char\* Population\_Manager::StateNames[100]

protected

Referenced by Skylark\_Population\_Manager::Init(), and SupplyStateNames().

unsigned Population\_Manager::StateNamesLength

protected

Referenced by SupplyStateNamesLength().

FILE\* Population\_Manager::TestFile

protected

FILE\* Population\_Manager::TestFile2

protected

vector< TListOfAnimals > Population\_Manager::TheArray

protected

Referenced by Skylark\_Population\_Manager::BreedingPairsOutput(), Skylark\_Population\_Manager::Catastrophe(), Skylark\_Population\_Manager::CreateObjects(), IsLast(), Skylark\_Population\_Manager::ProbePOM(), Skylark\_Population\_Manager::ReHouse(), Skylark\_Population\_Manager::Skylark\_Population\_Manager(), SupplyListIndexSize(), SupplyListSize(), SupplyLocXY(), SupplyState(), Skylark\_Population\_Manager::TheBreedingFemalesProbe(), Skylark\_Population\_Manager::TheBreedingSuccessProbe(), Skylark\_Population\_Manager::TheCIPEGridOutputProbe(), Skylark\_Population\_Manager::TheFledgelingProbe(), Skylark\_Population\_Manager::TheNWordOutputProbe(), and Skylark\_Population\_Manager::TheRipleysOutputProbe().

probe\_data\* Population\_Manager::TheProbe[100]

Referenced by CloseDownSim(), GetProbeInput\_ini(), and Skylark\_Population\_Manager::ProbeReportPOM().

---

The documentation for this class was generated from the following files:

- populationmanager.h
- PopulationManager.cpp


- Population\_Manager
- Generated on Thu Jan 10 2013 13:15:37 for ALMaSS Skylark ODdox by
   1.8.1.1
